# Minimizing the Risk of Catastrophic Health Expenditure in China: A Multi-Dimensional Analysis of Vulnerable Groups

**DOI:** 10.3389/fpubh.2021.689809

**Published:** 2021-08-06

**Authors:** Jiahui Wang, Xiao Tan, Xinye Qi, Xin Zhang, Huan Liu, Kexin Wang, Shengchao Jiang, Qiao Xu, Nan Meng, Peiwen Chen, Ye Li, Zheng Kang, Qunhong Wu, Linghan Shan, Daniel Adjei Amporfro, Bykov Ilia

**Affiliations:** ^1^Centre of Health Policy and Management, Health Management College, Harbin Medical University, Harbin, China; ^2^Department of Social Medicine, School of Public Health, Health Management College, Harbin Medical University, Harbin, China; ^3^Shenzhen Hospital of Guangzhou University of Traditional Chinese Medicine (Futian), Shenzhen, China

**Keywords:** catastrophic health expenditure, financial protection, universal health coverage, China, insurance

## Abstract

**Background:** In moving toward universal health coverage in China, it is crucial to identify which populations should be prioritized for which interventions rather than blindly increasing welfare packages or capital investments. We identify the characteristics of vulnerable groups from multiple perspectives through estimating catastrophic health expenditure (CHE) and recommend intervention priorities.

**Methods:** Data were from National Health Service Survey conducted in 2003, 2008, and 2013. According to the recommendation of WHO, this study adopted 40% as the CHE threshold. A binary regression was used to identify the determinants of CHE occurrence; a probit model was used to obtain CHE standardized incidence under the characteristics of single and two dimensions in 2013.

**Results:** The total incidence of CHE in 2013 was 13.9%, which shows a general trend of growth from 2003 to 2013. Families in western and central regions and rural areas were more at risk. Factors related to social demography show that households with a female or an unmarried head of household or with a low socioeconomic status were more likely to experience CHE. Households with older adults aged 60 and above had 1,524 times higher likelihood of experiencing CHE. Among the health insurance schemes, the participants covered by the New Rural Cooperative Medical Scheme had the highest risk compared with the participants of all basic health insurance schemes. Households with several members seeking outpatient, inpatient care or with non-communicable diseases were more likely to experience CHE. Households with members not seeing a doctor or hospitalized despite the need for it were more likely to experience CHE. Characteristics such as a household head with characteristics related to low socioeconomic status, having more than two hospitalized family members, ranked high. Meanwhile, the combination of having illiterate household heads and with being covered by other health insurance plans or by none ranked the first place. Cancer notably caused a relatively high medical expenditure among households with CHE.

**Conclusion:** In China, considering the vulnerability of the population across different dimensions is conducive to the alleviation of high CHE. Furthermore, people with multiple vulnerabilities should be prioritized for intervention. Identifying and targeting them to offer help and support will be an effective approach.

## Introduction

Throughout the 40 years of “reform and opening up” of China, the country has progressed remarkably in terms of socioeconomic development. Its GDP has increased from 368 billion yuan (USD 53 billion) in 1978 to 83 trillion yuan (USD 13 trillion) in 2019, witnessing an average annual growth rate of 15% (1). In addition to its economic progress, China has also accelerated its goal of improved universal health coverage (UHC). Notably, China set up the biggest medical insurance safety nets in the shortest time so as to cover as many people as possible. Since 2003, China has launched and implemented a series of basic medical insurance schemes, including Urban Employee Basic Medical Insurance (UEBMI), launched in 1998, which was provided mandatorily for employees in urban areas (also including retired and rural-to-urban migrant workers), whose premium is to be borne by both the employer and the employee, with a combined individual account and a socially pooled fund; individual accounts are mainly used for general outpatient services or to purchase drugs in the drug stores. New Rural Cooperative Medical Scheme (NCMS) was launched in 2003 as a voluntary system of mutual assistance through risk pooling to mitigate unaffordable health services and a financial burden in rural areas. The funding is from the contribution of an individual and the government; the Urban Resident Basic Medical Insurance (URBMI), launched 4 years after NCMS, was designed for urban residents not covered by UEBMI or NCMS, including primary and secondary school students, young children, and other unemployed urban residents. It is on a voluntary basis at the household level, and is sponsored by the government and an individual. Different from UEBMI, the individual accounts for URBMI or NCMS participants could not be used in the drug stores. Subsequently, integration of basic medical insurance systems was conducted in succession all over the country; the patterns of integrating URBMI and NRCMS (2) or integrated UEBMI, URBMI, and NRCMS were chosen according to their local conditions (3). Currently, the coverage rate of basic medical insurance is more than 95% (4). Great progress of China has also attracted international attention; a report published in the journal “The Lancet” evaluated and affirmed the progress of China in broadening insurance coverage, stating that Chinese insurance is the most extensive insurance program globally (5). The broad coverage of basic health insurance played a great role in alleviating the major financial barrier hindering smooth access to health services. The problem of seeking medical service, which Chinese often say, “Seeing a doctor is hard” has been improved to some extent (6). However, the strong performance in achieving extensive coverage was not sufficient to realize UHC, as the issue of affordability—“seeing a doctor is expensive”—still exists. UHC aims to ensure that no individual suffers financial hardship (7) when accessing quality health services. A 2010 World Health Organization (WHO) report interpreted UHC along three dimensions (8): breadth of coverage, that is, the proportion of the population that enjoys social health protection; depth of coverage, namely, the range of essential services necessary to effectively address health needs of people; and height of coverage, which refers to the portion of health-care costs covered through pooling and prepayment mechanisms (9).

Financial protection is one of the major aims of health systems (10) and has been, in general, captured by a well-established indicator called “catastrophic health expenditure” (CHE) (11, 12) and also, by the effects of out-of-pocket payments on poverty (13). According to WHO methodology, CHE is defined as out-of-pocket (OOP) spending for health care that exceeds 40% of a capacity of a household to pay (CTP) (14, 15). The CHE proportion in China has been at a high level; a study covering 133 countries revealed that China and some countries with inadequate health insurance coverage or in poverty were all listed at the most serious level (16). Unlike countries, such as Mexico, Thailand, and Vietnam, whose CHE rate has been falling with an increase in the proportion of the population with insurance coverage, the situation in China has not changed in the expected direction despite the launch of the three basic medical insurance schemes and the continuous expansion of coverage (16). According to the findings of the national survey in China, the CHE rate was up to 13% in 2008 and rose to 14.57% in 2012 (17, 18).

Since the health system reform was initiated in 2009, the Chinese government has made numerous efforts, increasing its investment in health care from 929.5 billion yuan in 2013 to 1445.1 billion yuan in 2017, with an increased rate of 55.5% (19). Among these, subsidies for medical insurance of urban and rural residents increased from 328.2 billion yuan to 491.9 billion yuan, at an average annual growth of 10.6% (19). A series of policies to alleviate CHE incidence were also launched, including zero markup policy on drug sales; more specifically, this policy aims to cancel the 15% drug markup when patients purchase drugs directly from hospitals and reduce the medical burden of patients (20), and establishing critical illness insurance. Despite the situation has improved, albeit not significantly, considering the limited available health resources, it is crucial to identify which populations should be prioritized for which interventions and consequently develop appropriate approaches to reach these targeted populations, as well as ensure appropriate allocation of resources and support, rather than blindly increasing welfare packages or capital investment. Besides, the actual situation of these vulnerable groups is masked by the average rate. It is difficult to address the economic burden of the vulnerable groups solely based on a generalized system of preferences. Being the main source of high CHE, improving the financial protection of vulnerable groups is the key to realize UHC, especially for developing or low-income countries (21).

Many studies have conducted research on CHE in China; however, only a few have reported national representative estimates for the whole population (17, 18, 22, 23). An in-depth analysis of vulnerable populations with high CHE risk is limited as well. Currently, China has just completed the task of eliminating absolute poverty, while the precise and quantitative identification of the mechanism of marginal groups that are prone to fall into poverty due to illness is still insufficient, with policymakers and the general public eager to know the progress of eliminating the risk of CHE in China and where its weakness lies (24, 25). This study examined the progress of China in enhancing financial protection, identifying the main characteristics of the high-risk population that need to be filled to fully achieve UHC. The results would also contribute to the development of healthcare systems in other nations with similar situations.

## Materials and Methods

### Study Design

This study conducted a comparative analysis between 2003 and 2013 to obtain the trend of CHE incidence and the changes of healthcare needs and health service utilization over time. An in-depth analysis was conducted on the fifth NHSS in 2013, identifying the factors with high risk and locking the characteristics with one risk factor, two overlapping risk factors after standardized.

### Data Source

Most data were obtained from the fifth National Health Service Survey (NHSS), which was conducted in 2013. Additionally, the data from the third and fourth NHSS were used for supplement. NHSS is a nationally representative survey organized by the Chinese government every 5 years. A multistage, stratified random sampling method was adopted in the NHSS to ensure a representative sample. All the participants were interviewed face-to-face by trained investigators; ultimately, 57,023 households in the third NHSS, 56,456 households in the fourth NHSS, 93,613 households in the fifth NHSS were included in the survey (26–28). After data cleaning, 57,023 households in the third NHSS, 56,433 households in the fourth NHSS, and 93,570 households in the fifth NHSS were respectively used in this study.

### Quality Control

The response rate of the fifth NHSS of the adult respondents was 82.1%. The test-retest reliability of the questionnaire reached 97.7%. In reference to the 2010 Sixth National Population Census data, the results show that there is no difference in the family size and the rural-to-urban household ratio between the sample of the fifth NHSS and the whole country; however, the proportion of older adults was higher in the sample than in the general population (26); the data of the third and fourth NHSS also show good consistency in household size between the surveyed population and the general population (27, 28).

### Measurement

#### Catastrophic Health Expenditure

According to the WHO definition, CHE occurs when the total OOP health payments of a household equal or exceed 40% of CTP of the household (15). OOP is the net after reimbursement under any type of insurance, including consulting fees of doctors, drug purchases, and hospitalization expenses, while excluding health-related transportation fees and special nutrition expenses. The CTP of the household is defined as the non-subsistence effective income of the household. The non-subsistence spending of the household was used as a proxy for CTP, and, when food expenditure was less than subsistence spending, CTP was defined as total expenditure minus food expenditure (15), while household subsistence spending was calculated as the poverty line multiplied by standard household size. The poverty line is defined as the food expenditure of the household whose food expenditure share of total household expenditure is within the 45th and 55th percentiles of the total sample (15).

### Variables

The dependent variable is whether a household experienced CHE. The independent variables included attributes concerning the household head, such as gender, education level, marital status, employment status, and insurance type; sociodemographic characteristics of households, such as region, economic quintiles (annual household consumption expenditure was ranked into quintiles after adjustment for standard household size), family size, and having family member over 60 years or younger than 5 years; and indicators on need and utilization of health services among household members, such as the number of people with non-communicable diseases (NCDs) in the last 6 months, or the number of people admitted in hospitals in the previous year; a preferred institution grade for common diseases and with members not seeing a doctor or hospitalized despite their need for it; the variables and their codes are detailed in Table 1.

**Table 1:** Basic information of respondents in the fifth NHSS.

**Variable**	**Variable value**	**Number (households)**	**Percentage (%)**
**Dependent variable**			
Experiencing CHE or not	0 = No	80,565	86.10
	1 = Yes	13,005	13.90
**Independent variable**			
Gender of the head of household	0 = Female	23,744	25.40
	1 = Male	69,826	74.60
Marital status of the head of household	0 = Married	78,751	84.20
	1 = Others	14,819	15.80
Educational level of the head of household	1 = Illiterate	9,904	10.60
	2 = Primary school	26,556	28.40
	3 = Junior high school	33,742	36.10
	4 = Senior high school and technical school	11,800	12.60
	5 = Technical secondary school and above	11,568	12.40
Employment status of the head of household	1 = Employed	64,026	68.40
	2 = Retired	16,164	17.30
	3 = Unemployed and students	13,380	14.30
Medical insurance type of the head of household	1 = UEBMI	23,919	25.60
	2 = URBMI	6,847	7.30
	3 = NCMS	43,362	46.30
	4 = Integration	12,060	12.90
	5 = Mixture	4,811	5.10
	6 = Others and none	2,571	2.70
Region	1 = Eastern	31,201	33.30
	2 = Central	31,186	33.30
	3 = Western	31,183	33.30
Location	0 = Urban	46,798	50.00
	1 = Rural	46,772	50.00
Household size	1 = ≤2	41,633	44.50
	2 = 3–4	39,289	42.00
	3 = ≥5	12,648	13.50
Household with members aged sixty and above	0 = No	52,792	56.40
	1 = Yes	40,778	43.60
Household with members aged five or younger	0 = No	77,239	82.50
	1 = Yes	16,331	17.50
Number of patients with chronic diseases	1 = 0	52793	56.40
	2 = 1	30,316	32.40
	3 = ≥2	10,461	11.20
Number of hospitalized members	1 = 0	74,641	79.80
	2 = 1	16,795	17.90
	3 = ≥2	2,134	2.30
Preferred institution grade for common diseases	0 = Primary hospital	75,833	81.00
	1 = Non-primary hospital	17,737	19.00
Whether there is a member go to clinic in 2 weeks	0 = No	75,006	80.20
	1 = Yes	18,564	19.80
Member not hospitalized despite the need for it	0 = No	89,516	95.70
	1 = Yes	4,054	4.30
Member not seeing a doctor despite the need for it	0 = No	66,440	71.00
	1 = Yes	27,130	29.00

### Data Analysis

Descriptive statistics were used to reveal the basic characteristics of the respondents and their healthcare needs and service utilization. A time trend approach was conducted to analyze the CHE incidence trend from 2003 to 2013. A logistic regression model was used to identify the determinants of CHE. The metrics for healthcare needs and service utilization and family population structure were standardized in the comparative analysis of the incidence of CHE across 31 provinces, a single-dimension factor, and two-dimension factors, other variables were used as control variables. A probit model was used to standardize the healthcare needs and service utilization (24, 29, 30). All statistical analyses were conducted, using STATA 11.0. Statistical significance was set at the 5% level.

## Results

### Basic Information Regarding Respondents of the Fifth NHSS

Most household heads were male (74.60%), married (84.20%), junior high school graduates (36.10%), and employed (68.4%). The NCMS was the most common medical insurance among household heads (46.9%). Nearly half of the households had members over 60 years old and members suffering from NCDs in the last 6 months; meanwhile, 44.5% of the households had no more than two family members (Table 1).

### Catastrophic Health Expenditure Incidence From 2003 to 2013

Table 2 shows the CHE incidences in 2003, 2008, and 2013, which were, respectively 11.7, 13.2, and 13.9%. The result of the time trend approach indicates that, from 2003 to 2013, the CHE incidence shows a general trend of growth over time (χ^2^ = 150.724, *P* < 0.001), although the growth rate slowed.

**Table 2:** Catastrophic health expenditure incidences in 2003, 2008, and 2013.

	**Total**	**Households with CHE**	**χ^**2**^**	***P*-value**
	***N***	***N* (%)**		
2003	57,023	6,649 (11.7)	150.724	<0.001
2008	56,433	7,445 (13.2)		
2013	93,570	13,005 (13.90)		

### Healthcare Needs and Service Utilization and Hospitalization Expenses From 2003 to 2013

We compared the healthcare needs and service utilization of the recent three NHSS conducted in 2003, 2008, and 2013. The results show that the healthcare needs increased over the years; the prevalence of NCDs was 33.1% in 2013, which was double than that in 2003 (15.1%). The non-admission rate, defined as the percentage of the respondents who had not been admitted to inpatient care in the past year despite being advised by a doctor, decreased from 29.6% in 2003 to 17.1% in 2013. The non-attendance rate, defined as the percentage of the patients who were ill but did not seek medical treatment in the past 2 weeks, decreased from 48.9% in 2003 to 27.3% in 2013.

**Figure 1:**
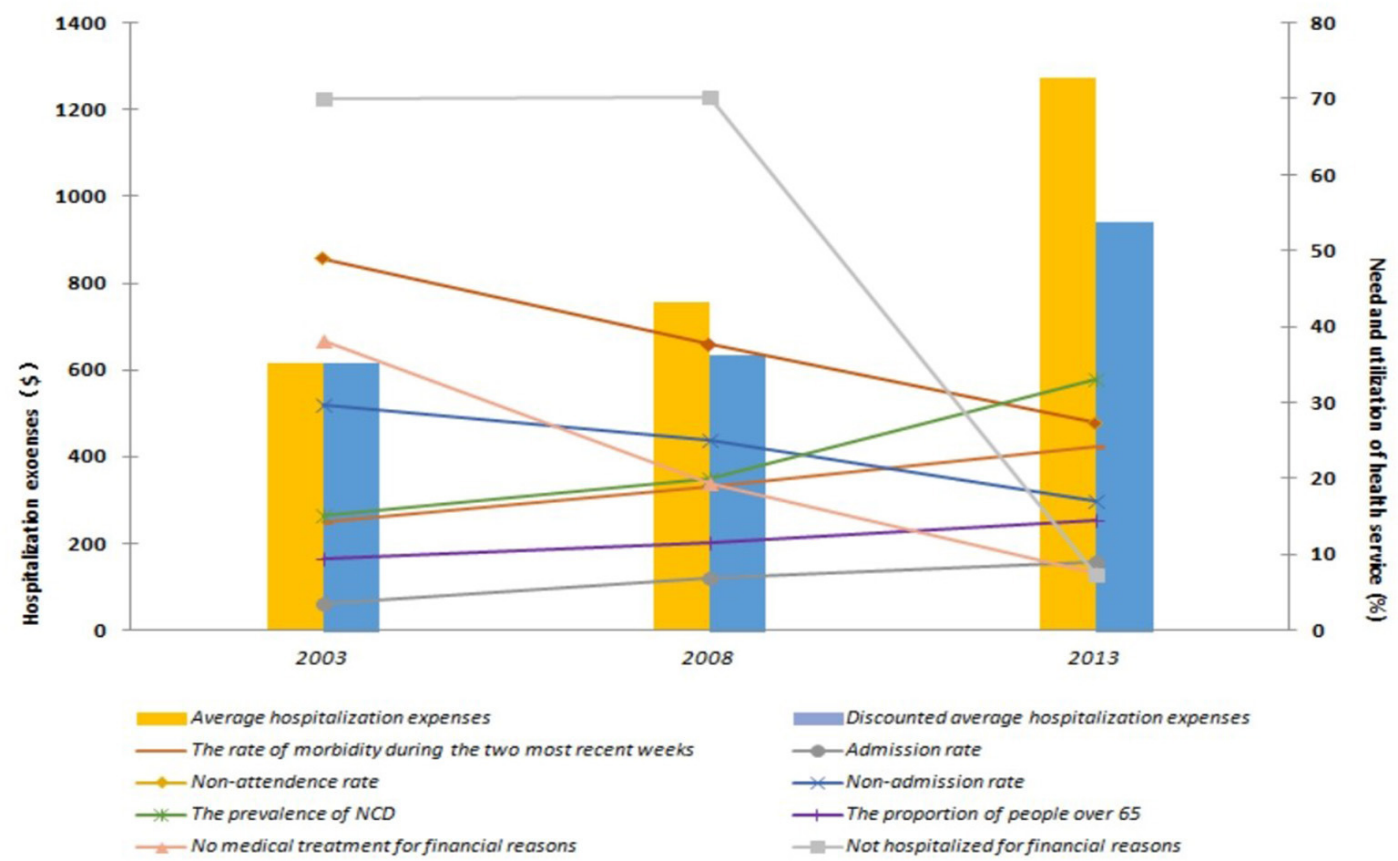
Comparison of health-care needs and service utilization in 2003, 2008, and 2013. $: USD, United States Dollar. According to the exchange rate of 6.1932 yuan to US$ 1.00.

Although the healthcare needs of residents have been met, this has resulted in higher medical expenses. Expenditure data for 2008 and 2013 are adjusted for movements in the consumer price index and taken 2003 as the basic year. After adjustment, through comparing the average hospitalization expenses of the three NHSS, it was found that the average hospitalization expenses increased over the years, from USD 616 (3,815 yuan) in 2003 to USD 1,273 (8,520 yuan) in 2013, with an annual growth rate of 19.9%. Furthermore, over the past decade, the average hospitalization cost increased by 106.6% (Figure 1).

### Multidimensional Analysis Based on Characteristics of CHE Risk Households

#### The Distribution of CHE Rate Among Regions and Provinces

**Figure 2:**
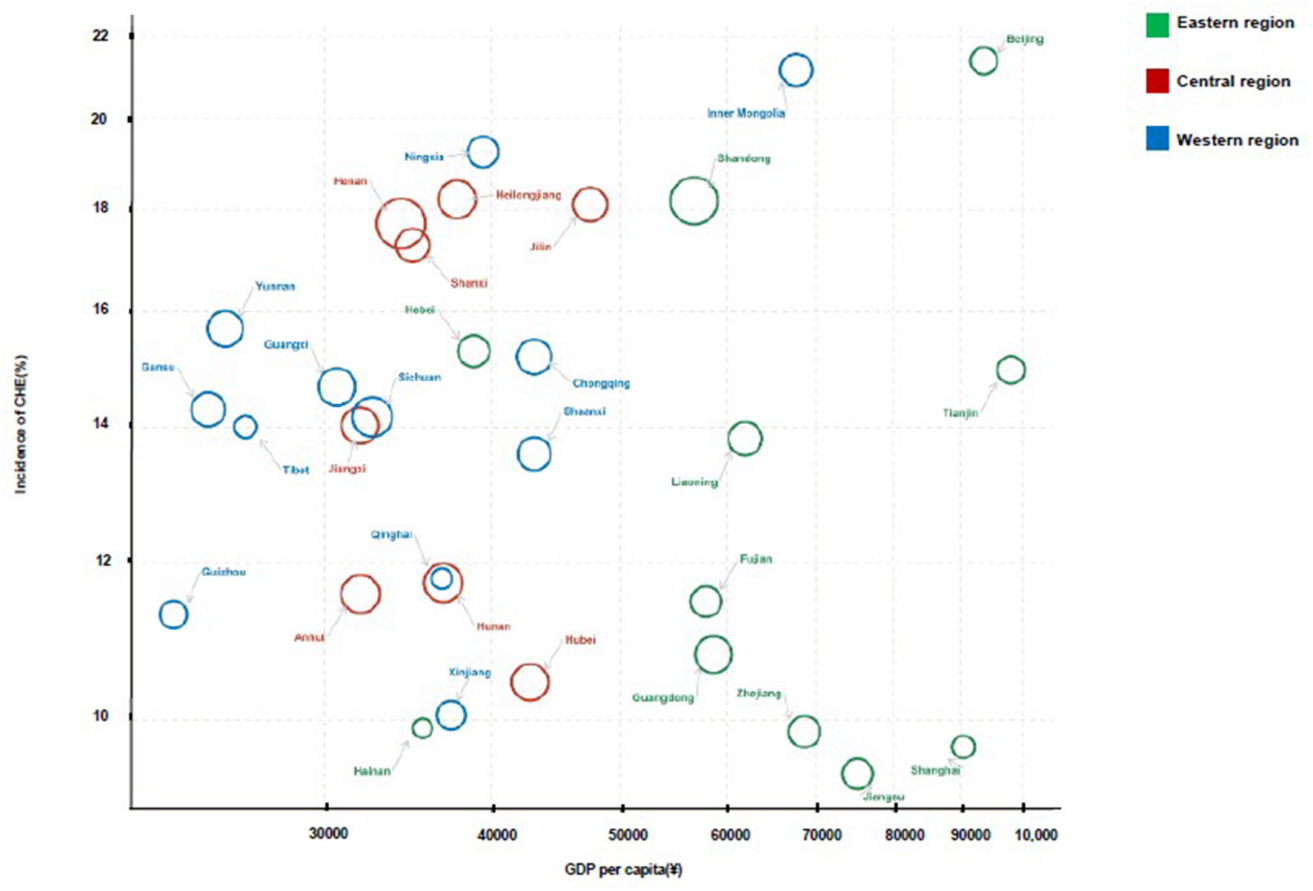
The incidence of CHE across 31 provinces in China.

The CHE proportion in the fifth NHSS was 13.9%. From a regional perspective, the proportion of CHE in rural areas is 1.2 times higher than that in urban areas; the central (14.4%) and western regions (14.3%) show a higher CHE rate compared with the eastern region (12.9%) (Table 3). Of the 31 provinces, 17 exceeded the national average level of the CHE rate, and most of them were concentrated in the central and western regions with lower *per capita* GDP. The number of households who suffered from CHE in the western region was larger than that in the eastern and central regions. Few cities such as Beijing and provinces such as Shandong had a fairly high CHE incidence (Figure 2).

**Table 3:** Univariate analysis of factors associated with CHE.

**Characteristics of the respondents**	**Total**	**CHE**	**Standard error**	**χ^**2**^**	***P*-value**
	***N* (%)**	***N* (%)**			
**Income quintile^a^**				766.742	<0.001
Quintile 1	18,714 (20.00)	3,703 (19.80)	0.0029		
Quintile 2	18,714 (20.00)	2,643 (14.10)	0.0025		
Quintile 3	18,714 (20.00)	2,369 (12.70)	0.0024		
Quintile 4	18,714 (20.00)	2,020 (10.80)	0.0023		
Quintile 5	18,714 (20.00)	2,270 (12.10)	0.0024		
**Household size**				2,379.113	<0.001
≤ 2	41,633 (44.50)	8,350 (20.10)	0.0020		
3−4	39,289 (42.00)	3,466 (8.80)	0.0014		
≥5	12,648 (13.50)	1,189 (9.40)	0.0026		
**Region**				33.961	<0.001
Eastern	31,201 (33.30)	4,046 (13.00)	0.0019		
Central	31,186 (33.30)	4,489 (14.40)	0.0020		
Western	31,183 (33.30)	4,470 (14.30)	0.0020		
**Location**				136.568	<0.001
Urban	46,798 (50.00)	5,886 (12.60)	0.0015		
Rural	46,772 (50.00)	7,119 (15.20)	0.0017		
**Gender**				24.709	<0.001
Male	69,826 (74.60)	9,476 (13.60)	0.0013		
Female	23,744 (25.40)	3,529 (14.90)	0.0023		
**Marital status of the head of household**				550.394	<0.001
Married	78,751 (84.20)	10,039 (12.70)	0.0012		
Others	14,819 (15.80)	2,966 (20.00)	0.0033		
**Educational level of the head of household**				2,327.951	<0.001
Illiterate	9,904 (10.60)	2,548 (25.70)	0.0044		
Primary school	26,556 (28.40)	4,691 (17.70)	0.0023		
Junior high school	33,742 (36.10)	3,867 (11.50)	0.0017		
Senior high school and technical school	11,800 (12.60)	1,120 (9.50)	0.0027		
Technical secondary school and above	11,568 (12.40)	779 (6.70)	0.0023		
**Employment status of the head of household**				2,635.196	<0.001
Employed	64,026 (68.40)	6,593 (10.30)	0.0012		
Retired	16,164 (17.30)	2,889 (17.90)	0.0030		
unemployed and students	13,380 (14.30)	3,523 (26.30)	0.0038		
**Medical insurance of the head of household**				404.361	<0.001
UEBMI	23,919 (25.60)	2,779 (11.60)	0.0021		
URBMI	6,847 (7.30)	986 (14.40)	0.0042		
NCMS	43,362 (46.30)	6,816 (15.70)	0.0017		
IBMIUR	12,060 (12.90)	1,774 (14.70)	0.0032		
Mixed medical insurance	4,811 (5.1%)	374 (7.80)	0.0039		
Other types and none	2,571 (2.7%)	276(10.70)	0.0061		
**Households including members aged above 60 years**				3,593.430	<0.001
No	52,792 (56.40)	4,192 (7.90)	0.0012		
Yes	40,778 (43.60)	8,813 (21.60)	0.0020		
**Households including members aged below 5 years**				395.525	<0.001
No	77,239 (82.50)	11,534 (14.90)	0.0013		
Yes	16,331 (17.50)	1,471 (9.00)	0.0022		
**Number of patients with chronic diseases**				4,182.198	<0.001
0	52,793 (56.40)	4,048 (7.70)	0.0012		
1	30,316 (32.40)	6,175 (20.40)	0.0023		
≥2	10,461 (11.20)	2,782 (26.60)	0.0043		
**Number of hospitalized members**				7,552.608	<0.001
0	74,641 (79.80)	6,711 (9.00)	0.0010		
1	16,795 (17.90)	5,415 (32.20)	0.0036		
≥2	2,134 (2.30)	879 (41.20)	0.0107		
**Preferred institutional grade for common diseases**				26.674	<0.001
Primary hospital	75,833 (81.00)	10,754 (14.20)	0.0013		
Non-primary hospital	17,737 (19.00)	2,251 (12.70)	0.0025		
**Whether there is a member go to clinic in 2 weeks**				1,254.815	<0.001
No	18,564 (19.80)	8,930 (11.90)	0.0024		
Yes	75,006 (80.20)	4,075 (22.00)	0.0015		
**Member not hospitalized despite the need for it**				1,443.631	<0.001
No	89,516 (95.70)	1,382 (34.10)	0.0016		
Yes	4,054 (4.30)	11,623 (13.00)	0.0053		
**Member not seeing a doctor despite the need for it**				1,875.437	<0.001
No	66,440 (71.00)	5,850 (21.60)	0.0016		
Yes	27,130 (29.00)	7,155 (10.80)	0.0019		

a *Quintile 1 is the poorest, 20%, and quintile 5 is the wealthiest, 20%*.

### CHE Incidence Cross Different Characteristics of Households

Table 3 shows the CHE incidences among households across different characteristics. The univariate analysis results show statistically significant relationships between CHE and factors related to the household head, demographics, and health service needs and utilization (Table 3).

### Determinants of Households Dropping Into CHE in 2013 in China

In the result of the model, *R*^2^ = 26.9%, and the Hosmer–Lemeshow test showed that the model fits well (*p*> 0.05), the logistic regression analysis revealed several drivers of CHE. Households in which the head was male (OR = 1.200, 95% CI = 1.138–1.264), unmarried (OR = 1.191, 95% CI = 1.124–1.263), or illiterate (OR = 1.191, 95% CI = 1.124–1.263) had a higher risk of CHE than other groups. Compared with households in the fifth quintile, households in Quintile 1 (OR = 1.483, 95% CI = 1.374–1.600) and Quintile 2 (OR = 1.241, 95%CI = 1.153–1.337) had a higher CHE risk. Compared with the household head with an education level of technical secondary school and above, the groups with an education level of illiterate (OR = 2.351, 95% CI = 2.114–2.616), primary school (OR = 1.779, 95% CI = 1.616–1.957), junior high school (OR = 1.510, 95% CI = 1.377–1.657), and senior high school and technical school (OR = 1.342, 95% CI = 1.208–1.491) were more likely to face CHE. Additionally, the better the employment status of the household head, the lower the risk of CHE in the household: compared with unemployed or student household head groups, employed (OR = 0.522, 95% CI = 0.493–0.552) and retired groups (OR = 0.756, 95% CI = 0.695–0.823) had a lower risk of CHE. Households without inpatient members (OR = 0.123, 95% CI = 0.111–0.136) or only one inpatient member (OR = 0.580, 95% CI = 0.523–0.643) were at a lower risk than those with more than two inpatient members. Compared with households with more than two NCD members, those with no NCD members (OR = 0.509, 95% CI = 0.473–0.548) and only one NCD member (OR = 0.842, 95% CI = 0.793–0.894) were at a lower risk of CHE. Households with outpatient members had a higher CHE risk than the reference (OR = 1.507, 95% CI = 1.433–1.584). Households in rural regions had a higher risk (OR = 1.062, 95% CI = 1.007–1.119) than urban households, while those in western (OR = 1.079, 95% CI = 1.023–1.138) and central regions (OR = 1.052, 95% CI = 0.998–1.109) had a higher risk than those in the eastern region. Households covered by UEBMI had the strongest ability to resist the economic burden of disease. Compared with UEBMI, other types of insurance revealed a higher risk of CHE, and, among these groups, the risk of NCMS was highest (OR = 1.508, 95% CI = 1.382–1.646). Notably, households with members not visiting a doctor (OR = 1.325, 95% CI = 1.256–1.396) or not hospitalized despite the need for it (OR = 1.757, 95% CI = 1.625–1.899) had a higher risk of experiencing CHE (Table 4).

**Table 4:** Determinants of CHE, using logistic regression.

	**Sig**.	**Exp(B)**	**95.C.I**.	
			**Lower**	**Upper**
**Income quintile**				
Quintile 1 vs. 5	0.000	1.483	1.374	1.600
Quintile 2 vs. 5	0.000	1.241	1.153	1.337
Quintile 3 vs. 5	0.125	1.058	0.984	1.137
Quintile 4 vs. 5	0.008	0.907	0.844	0.974
**Household size**				
≤ 2 vs. ≥5	0.000	4.000	3.682	4.344
3–4 vs. ≥5	0.000	1.854	1.712	2.008
**Region**				
Central region vs. Eastern region	0.061	1.052	0.998	1.109
Western region vs. Eastern region	0.006	1.079	1.023	1.138
Rural vs. Urban	0.025	1.062	1.007	1.119
**Gender of the head of household:** Female vs. Male	0.000	1.200	1.138	1.264
**Marital status of the head of household:** Others vs. Married	0.000	1.191	1.124	1.263
**Educational level of the head of household**				
Illiterate vs. Technical secondary school and above	0.000	2.351	2.114	2.616
Primary school vs. Technical secondary school and above	0.000	1.779	1.616	1.957
Junior high school vs. Technical secondary school and above	0.000	1.510	1.377	1.657
Senior high school and Technical school vs. Technical secondary school and above	0.000	1.342	1.208	1.491
**Employment status of the head of household**				
Employed vs. unemployed or student	0.000	0.522	0.493	0.552
Retired vs. unemployed or student	0.000	0.756	0.695	0.823
**Medical insurance of the head of household**				
URBMI vs. UEBMI	0.000	1.293	1.171	1.427
NCMS vs. UEBMI	0.000	1.508	1.382	1.646
Integrated medical insurance vs. UEBMI	0.000	1.316	1.195	1.448
Mixed medical insurance vs. UEBMI	0.337	0.938	0.823	1.069
Other types and none vs. UEBMI	0.003	1.256	1.080	1.459
**Having members aged≥60 years:** Yes vs. No	0.000	1.524	1.448	1.605
**Having members aged≤5 years:** Yes vs. No	0.162	0.949	0.882	1.021
**Members with chronic diseases**				
0 vs. ≥2	0.000	0.509	0.473	0.548
1 vs. ≥2	0.000	0.842	0.793	0.894
**Inpatients members**				
0 vs. ≥2	0.000	0.123	0.111	0.136
1 vs. ≥2	0.000	0.580	0.523	0.643
**Preferred institutional grade for common diseases**				
Non-primary medical institution vs. primary medical institution	0.065	1.059	0.997	1.126
**Whether there is a member go to clinic in 2 weeks:** Yes vs. No	0.000	1.507	1.433	1.584
**Members not hospitalized despite the need for it:** Yes vs. No	0.000	1.757	1.625	1.899
**Members not seeing a doctor despite the need for it:** Yes vs. No	0.000	1.325	1.256	1.396

### The CHE Standardized Rate Under the Characteristics of Single Dimension

**Figure 3:**
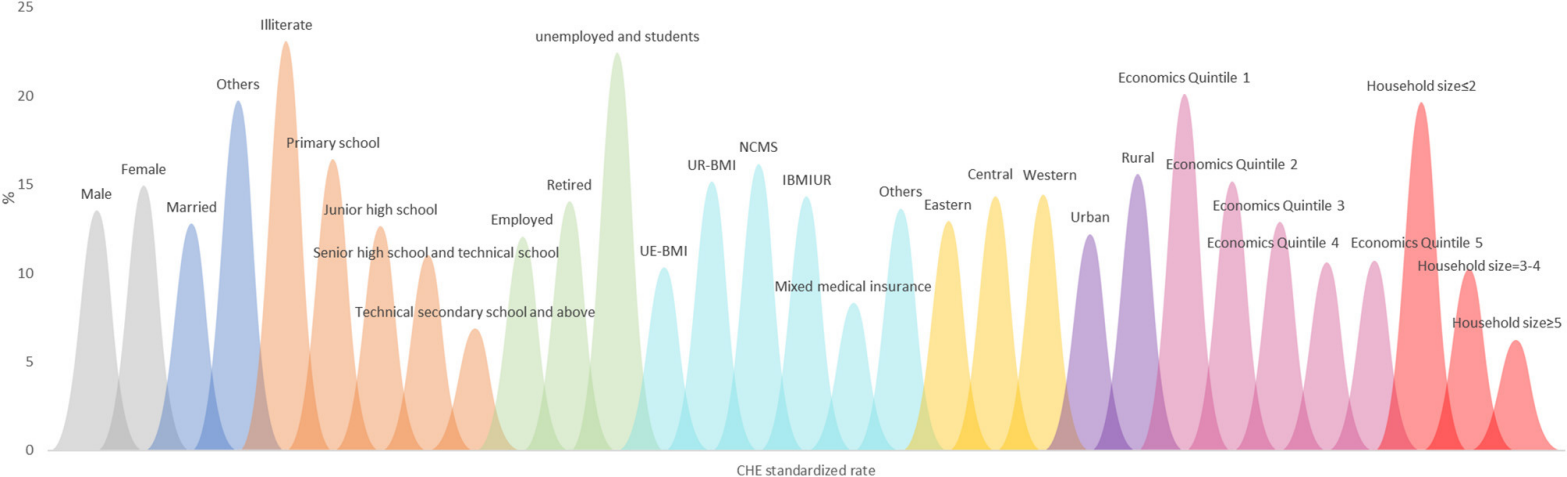
The CHE standardized rate under the characteristics of single dimension related to demographic factors.

**Figure 4:**
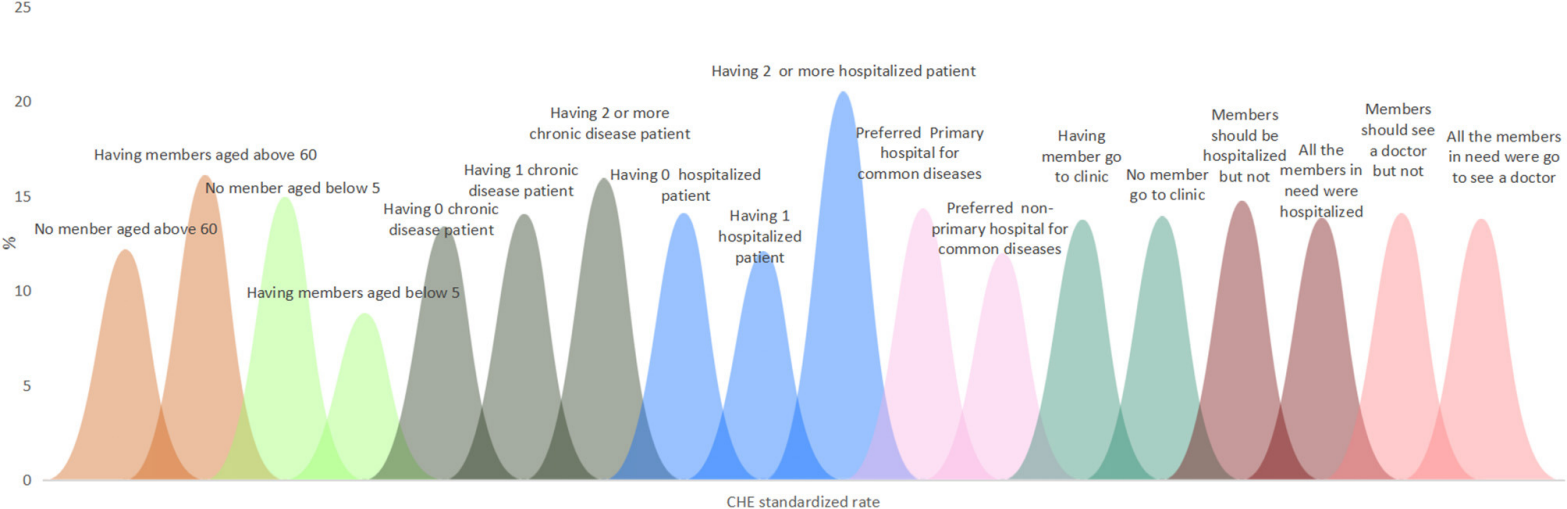
The CHE standardized rate under the characteristics of single dimension related to health service need and utilization factors.

Figures 3, 4, respectively, show the CHE standardized rate under the single dimension of demographic factors and health service needs and utilization factors. Regarding the demographic factors, the top three factors with the highest CHE risk were illiterate household head (23.06%), unemployed or student household head (22.43%), and the poorest household economic status (20.11%). Regarding the factors of health service need and utilization, the top three factors with the highest risk were having two or more hospitalized patients (20.53%), having two or more chronic disease patients (15.93%), and having members who should be hospitalized but are not (14.76%).

### CHE Risk Characteristics Under Different Location Levels, Region Levels and Economic Quintile Levels of Households, and Health Insurance Levels of Household Heads

**Figure 5:**
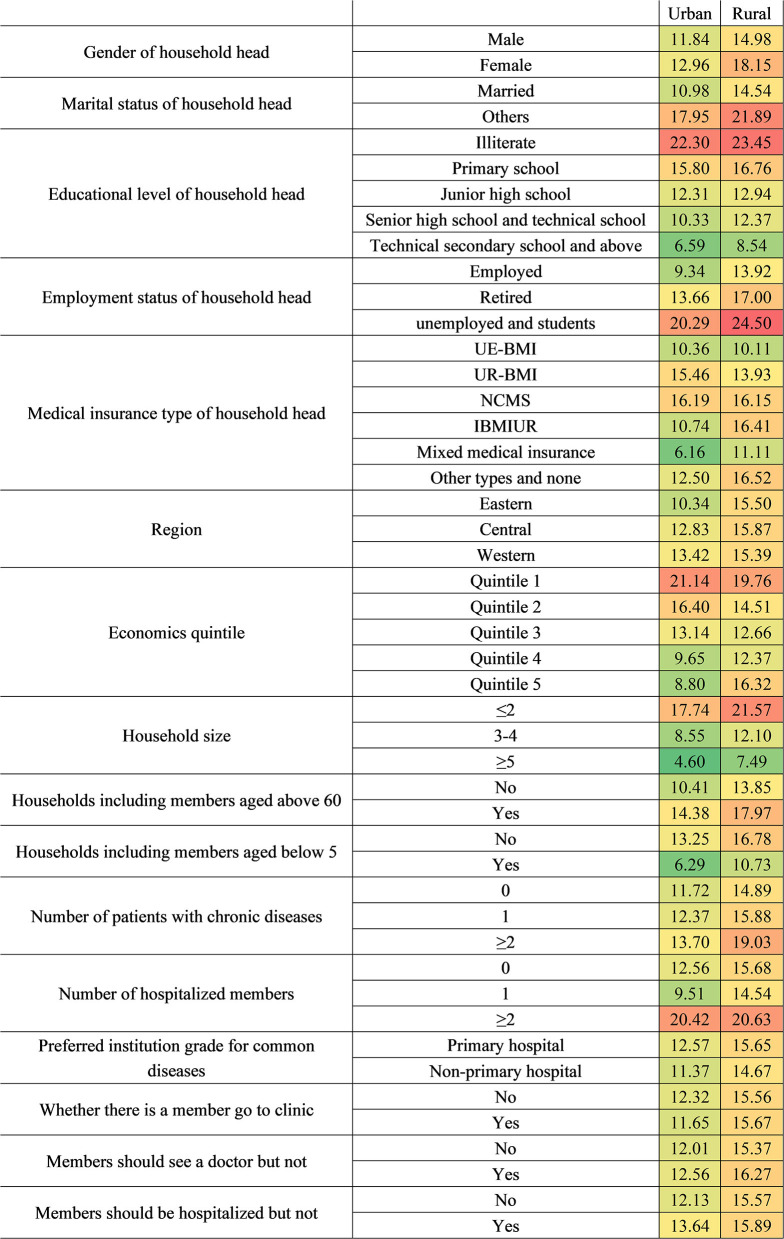
Factors affecting CHE by different locations of households.

**Figure 6:**
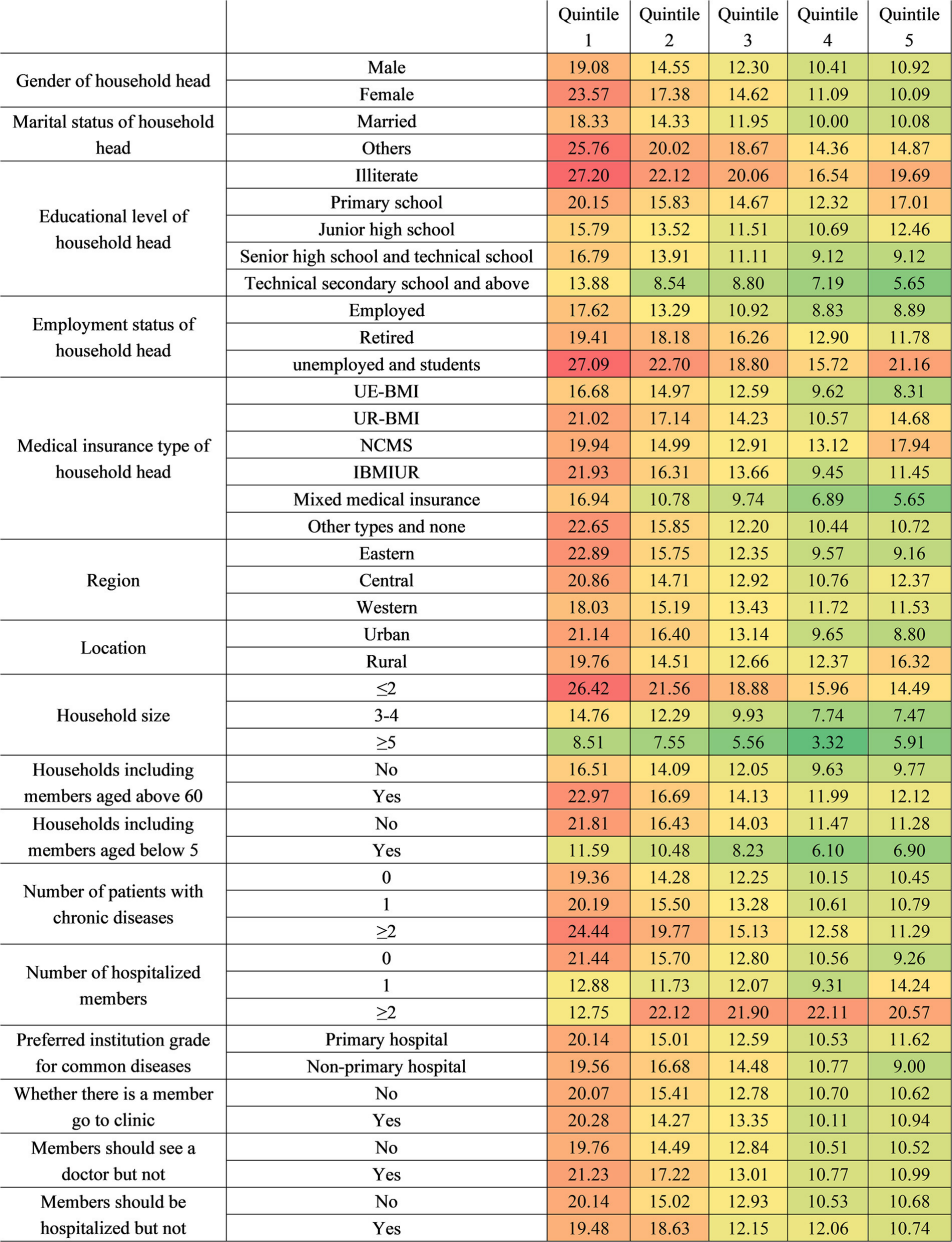
The CHE standardized rate by different economic quintile groups.

**Figure 7:**
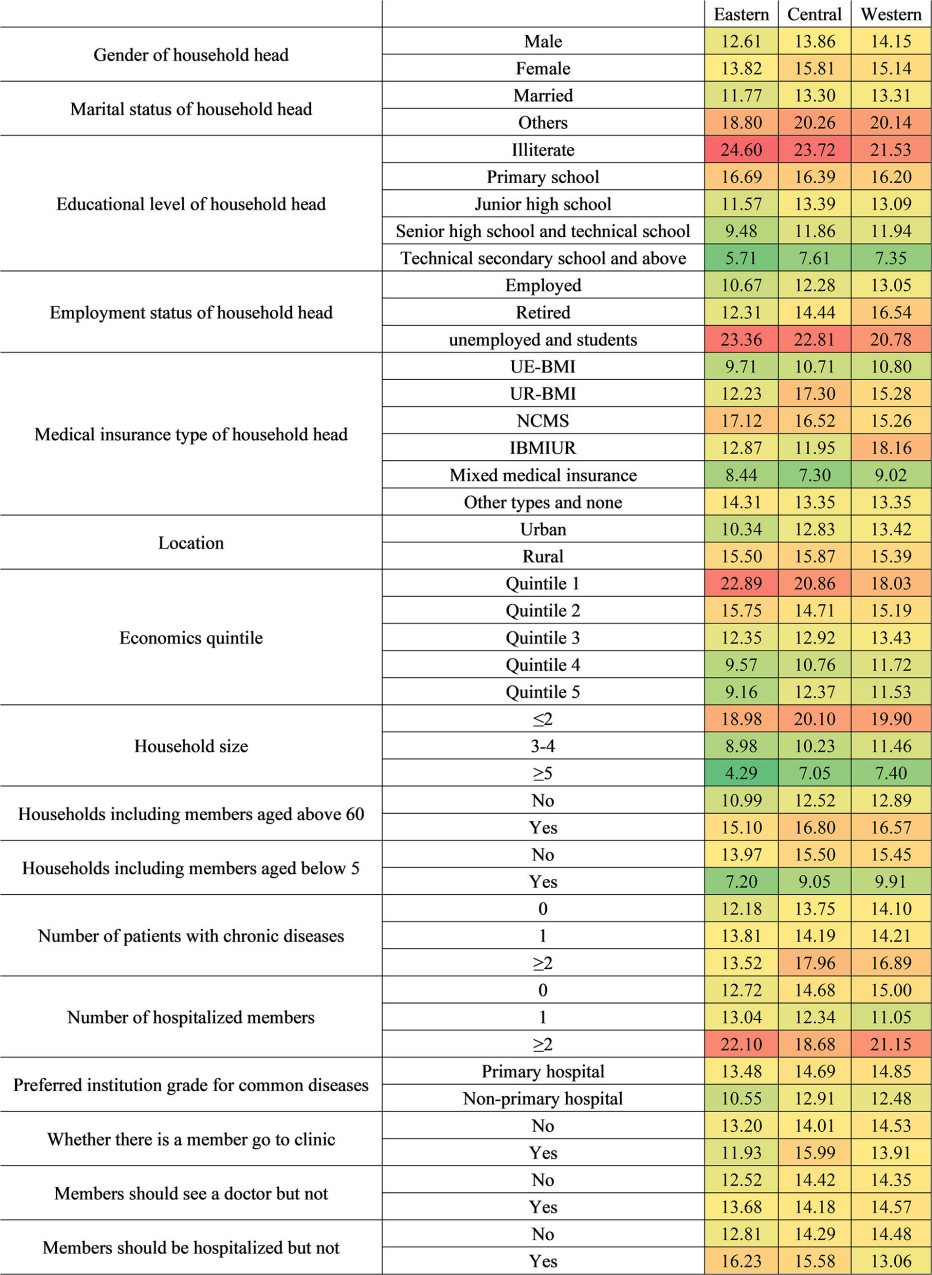
The CHE standardized rate by different areas.

**Figure 8:**
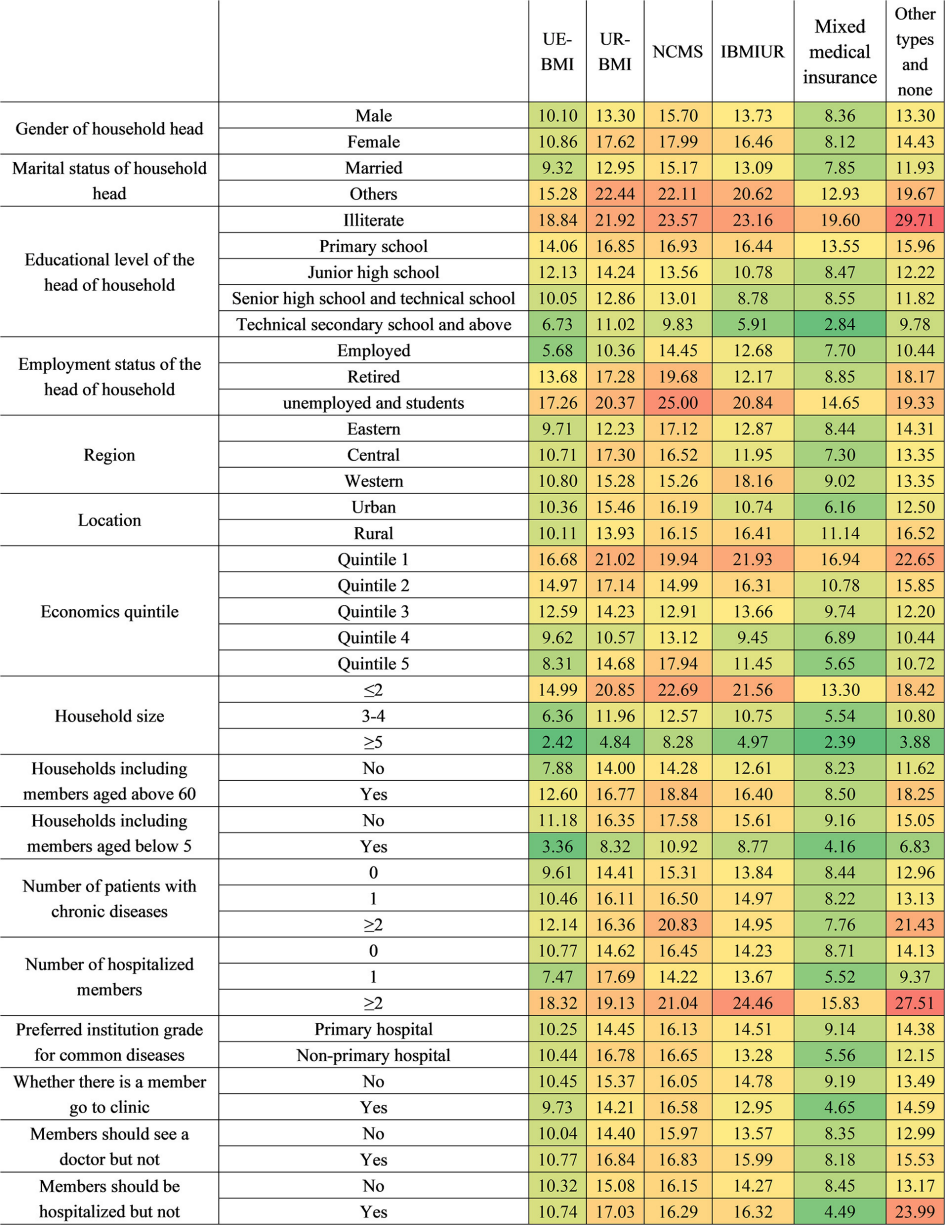
The CHE standardized rate by different health insurance systems. IBMIUR: Integration insurance refers to other basic medical insurance types, which integrate either URBMI and NCMS or UEBMI, URBMI, and NCMS. Mixture medical insurance: It refers to participants covered by basic medical insurance and commercial insurance at the same time.

Figures 5–8, respectively, show that CHE risk characteristics lied under different location levels and region levels and economic quintile levels of households, and health insurance levels of household heads. Regarding the location level of the household, the CHE rates of rural households were higher than urban households in general. Urban households with illiterate household heads have the greatest risk, with a standardized rate of 22.30%, followed by rural households with the poorest economic status (21.40%). In rural households, those with unemployed or student status household head ranked first (24.50%); those with an illiterate status also had a considerable CHE risk (23.45%).

Regarding household region, eastern, central, and western households with illiterate household heads were all the most vulnerable. In addition, unemployed or student status was also a risk characteristic for households from three regions. The CHE risks of eastern and western households with more than two hospitalized members were quite high at 22.10 and 21.15%, respectively. The poorest households located in eastern and central regions also need to be paid attention to meanwhile.

Regarding economic status, the poorest households with different characteristics show higher CHE rates than other economic groups at over 20%. In the poorest households, those with illiterate household heads ranked first (27.20%), followed by those with unemployed or student household heads (27.09%). For households with sub-poorest economic status, those with household heads who were unemployed or students were the most vulnerable (22.70%). Notably, the richest group with unemployed or student status household head also had a considerably high CHE incidence (21.36%). With the exception of groups in Quintile 1, economic groups with more than two hospitalized members had considerably high CHE risk.

Regarding the health insurance level, households with UEBMI or mixed medical insurance had a lower CHE risk than households with other insurance types. Household heads covered by URBMI with an unstable married relationship were the most vulnerable group (22.44%), followed by those who were illiterate (21.92%). For NCMS households, household heads who were unemployed or students (25.00%) ranked first, and illiterate household heads (23.57%) ranked second. For IBMIUR households, those with more than two hospitalized members had the highest CHE rate (24.46%), and the integrated insurance category shows a lower CHE rate than URBMI or NCMS; the lower rate was largely due to the lower CHE rate among urban residents, as the value among rural residents was higher. For those with other insurance types or those without medical coverage, the top two risk characteristics were having an illiterate household head (29.71%) and having more than two hospitalized members (27.51%).

### Top Five Characteristics of Single or Two Dimensions in Occurring CHE

In order to determine targeted interventions, our analysis listed the top five characteristics of households with a single risk factor and two combined CHE risk factors. In the top five characteristics of households with one risk factor, we found that households whose household heads were illiterate, unemployed or students, whose number of inpatient members was no more than two occupying the top three (23.06, 22.43, 20.53%). Next were households with the poorest economic status and household heads in unstable marriages. Regarding the top five characteristics of households with two combined risk factors, those with an illiterate household head and who were covered by other health insurance types or not covered ranked first, followed by household size was no more than two, combined with the household heads, who were illiterate, and household size was no more than two and the household heads were unemployed or student status (Table 5).

**Table 5:** Top 5 family characteristics related to CHE with a single risk factor and two combined CHE risk factors.

**Order**	**Top five types of families with one risk factor**	**CHE standardized rate (%)**
1	Illiterate household head	23.06
2	Household head with unemployed and student status	22.43
3	Household with 2 or more hospitalized members	20.53
4	Poorest households	20.11
5	Household head with unstable marriage	19.72
**Order**	**Top five types with two combined risk factors**	**CHE standardized rate (%)**
1	Illiterate household head covered by other health insurance types or not covered	29.71
2	Household size was no more than 2 and the household head was illiterate	29.59
3	Household size was no more than 2 and the household head was unemployed or a student	29.25
4	Household with more than two inpatient members and the household size was no more than 2	28.76
5	Household head was illiterate, unemployed, or a student or household had more than 2 inpatient members and an unemployed or student household head	27.92

### Top 10 Diseases of Total Hospitalization Expenses of Households With CHE

This study analyzed the top 10 diseases with the highest total hospitalization expenses for households experiencing CHE. There were four diseases for which the total hospitalization expense exceeded 1,766.14 USD and the highest being 2,099.08 USD for a malignant tumor, congenital heart disease or other congenital abnormalities, and NCDIs. Notably, among the top four diseases, the number of CHE cases caused by a malignant tumor is the highest (768 households), followed by injury and poisoning (753 households) (Table 6).

**Table 6:** Top 10 diseases with the highest total hospitalization expenses of households with CHE.

**Order**	**Disease**	**Number (households)**	**Total hospitalization expenses (USD)**
1	Malignant tumor	768	2,099.08
2	Congenital heart disease or other congenital abnormalities	18	2,099.08
3	Benign tumor, tumor *in situ*	204	1,776.14
4	Injury and poisoning	753	1,776.14
5	Urogenital system disease	516	1,291.74
6	Mental disease	60	1,178.71
7	Infectious disease	101	1,130.27
8	Disease of blood and blood forming organ	74	1,130.27
9	Neurological disease	174	1,114.13
10	Other diseases	146	1,106.05

*USD, United States dollar, according to the exchange rate of 6.1932 yuan to USD 1.00*.

## Discussion

The unrelentingly high CHE rate in China has not improved despite the efforts of the Chinese government. It is difficult to achieve the expected effect by increasing welfare and compensation policies indiscriminately, without considering population targeting. This study estimated the CHE rate at the geographic levels (national, regional, and provincial), in addition, exploring factors related to demography, disease, health service need, and utilization to delineate the key risks and vulnerable populations that would help develop dynamic and effective responses aimed at those families experiencing CHE. The analysis shows that the total CHE rate in 2013 (13.9%) was 6.92% higher than that of the previous NHSS in 2008; however, the growth rate was lower than that between 2003 and 2008. Households located in rural areas, in the western region, with members who are NCMS participants, with members over the age of 60, with a female household head or a household head, with low socioeconomic status or unstable married relationship, and with smaller household size, more hospitalization, outpatient, and NCD members were more vulnerable to CHE. In the ranking of single risk factors for CHE, the top five factors leading to the highest CHE incidence were primarily concentrated in households with low socioeconomic status or having more than two inpatient members. Meanwhile, in the overlapping risk factors for CHE, the combination of having illiterate household heads and being covered by other health insurance plans or by none ranked the first.

Compared with other developing countries, the CHE incidence rate in China is relatively high (31). A series of estimates from other countries around 2013 applied 40% as the CHE threshold and found that the CHE incidences of Mongolia and Kenya were lower than that of China, at 1.1% (31) and 4.52% (32), respectively; meanwhile, CHE incidence of Nepal is similar to that of China at 13.8% (33), while that of India is higher than that of China at 18.2% (34).

The increasing economic burden was attributed to several aspects; on the one hand, the aging population and its health service needs, especially the prevalence of chronic NCDs, increased rapidly, leading to a rapid growth of health expenditure (35). On the other hand, the improvement of economic status, the development of medical technology, and the universal coverage of the medical insurance system promoted the utilization of health services. In our study, the hospitalization rate rose to 9%, and the proportion of people giving up treatment or hospitalization for financial reasons decreased to 7.6 and 7.4%, respectively. In the case of surging health service needs, health insurance, in turn, largely stimulated the utilization of health services, thereby leading to higher CHE (which occurs when health insurance does not provide adequate financial protection). Thus, the improvement of the benefits package, reasonable reimbursement standard setting, and effective payment mechanism to control health expenditure were especially crucial to alleviate the risk of CHE and achieve UHC. This study aimed to identify the vulnerable groups and provide targetable strategies that should be prioritized. The study conducted an analysis of the characteristics of multiple vulnerable groups across the following levels.

### Households Located in the Western Region, Living in the Rural Areas, Were More Easily to Drop Into CHE, the High CHE Rate, Owing to Overutilization in Developed Regions Should Be Paid More Attention

From the perspective of the region, the central and western regions had higher CHE incidence risk than the eastern region, with 13.4 and 13.3%, respectively. It is worth noting that, in the case of no statistical difference between the central and western regions on the CHE rate, the hospitalization rate in the central region (8%) is lower than that in the western region (8.6%), but the average hospitalization expenses were higher in the central region (Supplementary Material 1) (26). This illustrates the regional differences in the design of medical insurance schemes lead to different patterns of an economic burden. Through the analysis of CHE proportion in 31 provinces distributed in the eastern, western, and central regions, we found that the CHE rate of most provinces in the eastern region was below the national average and was also generally lower than that of the central and western regions, reflecting that a better economic level contributed to resisting CHE. However, another finding indicated that there exist some eastern provinces with high GDP *per capita* with considerably high CHE rates, such as Beijing, Shanghai, and Tianjin Province, revealing that, when the economic development reaches a certain extent, it results in higher CHE incidence instead, owing to utilization (or even overutilization) of health services. Moreover, a high degree of a gap in financial protection between rural and urban residents still exists. Our study shows that the CHE rate in rural areas is associated with a higher CHE proportion, and rural residents had 1.062 times higher risk than urban residents. Another study also confirmed that the residents in rural areas suffered a greater financial burden from health expenditures; the percentage was 2.4 times higher than that of the urban residents in 2013 (36).

### Households With Smaller Household Size and Unstable Married Relationships Were Vulnerable; Medical and Nursing Services for the Older People Would Increase Several Times Risk of CHE for Their Families

Our study shows that the larger the household size was, the lower the possibility of CHE risk was; the likelihood of experiencing CHE in the households with less than two members was ~4 times compared with the households with more than five members. More family members implies better risk-sharing ability and more working members entering the labor market, and, in turn, this reduces the risk of the household experiencing CHE. A recent study shows that, as the number of household members increases, the risk of impoverishment by medical expenses is reduced by 1.5% points (37). At the same time, we found that marriage is a protective factor, illustrating that existence of stable married relationships in a household is more conducive to reducing the possibility of CHE. The results also indicated a significantly higher probable connection between CHE incidence and households with older adults (OR = 1.524, 95% CI = 1.448–1.605), as reported in other studies in China (17, 22, 38); this is consistent with the results reported by Yardim et al. in Turkey (39) and Kavosi et al. in Iran (40). The reasons for high medical expenses among older adults were listed as follows: first, most older people suffer from NCDs of varying degrees; however, health services and drugs covered by health insurance policies for NCD in clinics were limited (41); thus, they either paid high proportion OOP spending for it or seeking inpatient service, which need not have been carried out, just for reimbursement (42, 43). In addition, during a period of recuperation or self-treatment at home, self-purchased drugs from drugstores are a crucial support for older people. As such purchases were made outside medical institutions, most families covered by insurance other than UEBMI have to bear this part of the high costs alone. Outside medical services, additional nursing expenditures for older adults are also a considerable economic burden, and this is an increasing problem for vulnerable households supporting older adults in China (44). In fact, these costs do not belong to the scale of OOP; thus, the financial risk of households with elderly members was conservatively undervalued in our study. Although the Chinese government has established long-term insurance for those with care needs, the scope of policy implementation is limited to pilot areas, and the policy remains in the pilot stage. Similar in many ways to China, Kenya has also established supplementary insurance to better economically protect the elderly, although this coverage is relatively limited (45). A previous study on calculating a long-term insurance pilot indicated that implementing long-term care insurance may effectively reduce hospitalization and outpatient expenses. Thus, pilot projects should be accelerated and extended to the whole population as soon as possible.

Meanwhile, we must seriously consider the aging population, the level of aging, until this study was conducted, had reached 202 million, and the number is projected to double by 2040 (343.8 million) (46, 47). It is estimated that the healthcare expenditure for Chinese older adults will almost double over the next three decades, which will be up to 263 billion CNY in 2050 (48). Thus, the strategic and tactical reserve for dealing with the aging society should be completed in this period. On one hand, we should implement a positive population policy, promoting relevant economic and social policies to match the current fertility policy to delay the aging process, and, on the other hand, we must strengthen the health management for older adults by establishing health records and providing free physical examination every year to control or delay the development of diseases.

### Households With Lowest Capacity to Pay Should Be Provided More Powerful Financial Protection in Medical Health Insurance; A Longer-Term Approach Is Improving the Education Level of the Disadvantaged Families

We found that superior socioeconomic status (a higher household economic level, a higher education level of a household head, and employed status of household heads) could reduce the odds of experiencing CHE and *vice versa*, which has been comprehensively prove in many studies (17, 22). Our study notably found that only the poorest (OR = 1.483) and the sub-poorest (OR = 1.241) groups show significantly higher risk than the richest, while some previous studies from low- and middle-income countries show a different association between CHE risk and economic status. In India, the poorest households tend to forgo healthcare that might bring them financial hardship, and, thus, relatively poor households are associated with a higher incidence of CHE (33). In Thailand, essential health services are sufficiently covered by social insurance, and the utilization of private high-level health services, resulting in the high CHE rate is more likely to occur in high income populations (49, 50). On the contrary, in China, there is an established health insurance system, covering more than 95% of the population. Health service access, especially among the low-income group, has greatly improved; however, the reimbursement rate is not sufficiently high to protect people from high OOP payments for essential health services (17). In this context, the participants did not have to forgo healthcare services, but as the reimbursement rates of their insurance systems were not sufficient, the lower-income groups were more likely to be associated with a higher incidence of CHE.

In addition, we found that households with household heads who were illiterate or unemployed or students always ranked the highest in each level. This illustrated that, although providing subsidies could alleviate temporary poverty, long-term and radical planning should pay more attention on improving education or providing better employment opportunities, as it is the only way to hinder the intergenerational transmission of poverty (51).

### Households Covered by NCMS Had the Highest CHE Risk; the Integration Reform of Basic Health Insurance Was Conducive to Improve Its Financial Protection

Our study shows that in all medical insurance types—URBMI, NCMS, and integrated insurance—the participants show higher CHE incidence than UEBMI, which are 1.1, 1.2, and 1.0 times higher, respectively, than the national level. The participants of NCMS were the most vulnerable, followed by those covered under URBMI, while the integrated insurance participants show a lower rate than those of URBMI or NCMS, indicating that the integrated social health system was probably conducive to provide more financial protection to the beneficiary. However, the integrated insurance participants do not seem to show greater advantages in terms of reducing CHE risk as expected. As of this study, a few districts in China pioneered the integration of the rural and urban schemes and, in an even smaller number of districts (Dongguan and Zhongshan), directly merged the UEBMI, URBMI, and NCMS (52).

Meanwhile, we found that the integrated reform only benefited urban residents, whereas the CHE occurrence among rural residents under integrated insurance is higher than non-integrated insurance. Several studies also proved that, under the integrated insurance, the urban and rural participants experience a larger gap in financial protection (53, 54), and the disparity was caused by a lack of consideration for the propensity of rural residents in medical insurance financing and health service utilization.

Under the integrated insurance, former fixed-point medical institutions were unified under a bigger management scope, and, hence, improvement in convenient off-site medical billing of integrated insurance may increase cases of rural patients going to urban hospitals. Increasing hospitalization rates in tertiary medical institutions and decreasing hospitalization rates in primary and secondary institutions were witnessed in western China after integration, which supports the findings of this study (55). Furthermore, high-quality medical resources are mostly concentrated in urban areas (56, 57), even as rural residents are prompted to use health services and, therefore, enormously increase their medical costs. Among the nationwide financing modes of integrated insurance, most of them have not transformed several financing levels to only one financing level. The problem with setting several financing levels is that rural residents tend to choose a lower financing level, whether limited by income or influenced by past habits (58, 59). However, urban residents tended to receive more health services and enjoy better welfare. This leads to a reverse subsidy (60, 61) and, thus, results in a new inequality. Therefore, in areas with a large gap in urban and rural economic development, the financing subsidy should be provided for rural residents.

### Strengthen Financial Protection for Cancer Patients and Ensure Insurance Coverage of Commonly Used Anticancer Drugs and Their Accessibility

Demand and utilization of health services were the necessary conditions for health expenditure. Our results indicated that households with NCD members faced a higher CHE risk. Our analysis of the top 10 diseases for which people in households with CHE were hospitalized revealed that the households with members with a malignant tumor or congenital heart disease or other congenital abnormalities suffered the highest expenses (2,099.08 USD); however, the majority of households (768) experienced CHE due to a malignant tumor. Malignant tumors were found to be a huge challenge not only because they can be life-threatening but also because they have high likelihood of leading to CHE (62). A calculation of cancer costs in different countries shows that, in the US, the proportion of household expenses for cancer patients was only 20.9%, while in China it was as high as 78.8% (63). Many anticancer drugs were not covered in the reimbursement scope or the reimbursement ratio was not so high, and these were the main reasons for high medical costs. Since 2018, the Chinese government has gradually included anticancer drugs in its medical insurance catalog through negotiation; however, barriers related to anticancer drugs arriving at hospitals remain and must be overcome to solve the imbalance in the demand and supply of drugs (64) and enable patients to enjoy the benefits of the policy. Meanwhile, support for chronic disease prevention and cancer screening must also be strengthened.

### Pay More Attention to Households With Several Patients and Remove the Obstacles to Treatment Delay

Several previous studies have shown that having inpatient or outpatient members in the household increased the probability of CHE (65). In our result, having more than two hospitalized members was the highest CHE risk factor of the health service and utilization factors. It is necessary to establish a family-based identification mechanism for vulnerable groups in the medical system and provide targeted additional fee relief or subsidies for them.

A surprising finding was that household members who should see a doctor or be hospitalized but had not done so experienced 1.757- or 1.325-times higher risk, respectively, compared with the reference group. One possible explanation was that, because of forgoing medical services that they should have been received, the treatment opportunity was greatly delayed, thereby increasing future medical expenses. Although the value in 2013 is much lower compared with the last survey in 2008, the proportion of those not hospitalized due to financial reasons reached nearly 50% (Supplementary Material 2). Thus, the government should make efforts to ensure that all the poverty groups are covered by basic and catastrophic health insurance schemes, appropriately decrease the deductible, increase the cap on hospitalization reimbursement expenses, and increase the reimbursement proportion within the policy scope. Furthermore, the government should take steps to enhance medical assistance to the poverty groups, such as decreasing the thresholds of medical assistance and expanding the medical assistance benefits scope (66). It is necessary to strengthen their regular monitoring to prevent them from returning to poverty.

### Priority Intervention Targets and Suggestions

The analysis of characteristics of households with single dimension risk factors for CHE showed that families with factors related to low socioeconomic status ranked a high place; households with more than 2 hospitalized members and those with unstable marriages were also among the top five characteristics. Therefore, these three categories of vulnerable groups in the low socioeconomic-status group are primary concerns. High costs of hospitalization are mainly due to excessive medical treatment and unreasonable hospitalization behavior (24). Thus, measures should be taken for reforming payment mechanism and improving the medical insurance policy for out-patient care to control hospitalization payments, especially for NCDs. Increasing investment in prevention of NCDs and management of NCD patients should also be seriously considered. It is difficult for households with an unemployed household head to resist the risk of CHE, and, thus, policy support is crucial for them. When two risk factors—illiterate household head and lack of health insurance—are combined, the CHE incidence increased to nearly 30%. This is followed by the combinations of household size ≤2 and illiterate household head, household size ≤2 and employed household head. The disadvantaged groups tend to have multiple vulnerabilities. Identifying the groups with multiple vulnerabilities and taking relevant measures would be an ideal approach to address the persistently high CHE rate. However, capturing the groups with multiple vulnerabilities depends on the degree of realization of household information as well as filing and data sharing among different departments at the national level.

The disadvantaged groups tend to have multiple vulnerabilities. Identifying the groups with multiple vulnerabilities and taking relevant multifaceted measures would constitute an approach to address the high CHE rate, which has been high for a long time. However, capturing the groups with multiple vulnerabilities was up to the realization degree of information filing and data sharing of households among different departments at the national level to a large extent.

The study had two distinctive strengths. First, the data used in this study were obtained from a nationally representative survey organized by the Chinese government. This survey, which included a large-scale sample undertaken with a multistage, stratified random sampling method, reflects the situation of CHE incidence and expenditure accurately. Additionally,we conducted a standardized calculation on the CHE rate under single dimension and two dimensions risk characteristics of Chinese households. The metrics for healthcare needs and service utilization and population structure of a family were standardized in the comparative analysis of the incidence of CHE across 31 provinces, a single-dimension factor, and two-dimension factors. There were also several limitations in our study. First, this was a cross-sectional study; thus, the causal relationship between predictors and CHE is not reflected. Second, the data were self-reported by the respondents, and there may be recall bias in the responses. Third, owing to some respondents being unable to afford health service payment, it might lead to the underestimation of CHE to some extent. Fourth, the data used in this study have their limitation as they restrict this study before 2013, owing to the lack of data availability after that. Fifth, the CHE rate was estimated, using only the ATP approach with the threshold of 40%. Using the definition of 10% of an income (or its proxies) threshold as the SDG use would be more comprehensive.

## Conclusions

This study investigated and identified the high CHE incidence group from the perspective of the region, family structure, socioeconomic status, medical insurance, and needs and utilization of health services. We found that from 2003 to 2013, the CHE incidence shows a general trend of growth over time and the incidence in 2013 was also higher than most developing countries. Households located in western regions and rural areas were more vulnerable, and several provinces located in the eastern regions also had a considerably high CHE incidence, owing to overutilization. Households with smaller size, lower socioeconomic status, unstable married relationship of household heads or members aged over 60 had higher CHE risk. Household heads covered by NCMS had the highest risk of CHE, and, while the integrated insurance was conducive to provide more financial protection, it performed worse among rural residents. Households with more NCD members or inpatient members had higher CHE risk. And, among these types of NCDs, the number of families with CHE due to cancer was the largest, and their total medical expenditure is the highest. Households with hospitalized members or with members not seeing a doctor /hospitalized despite the need for it increased the likelihood of CHE. Households with characteristics related to low socioeconomic status, having more than two hospitalized family members, were the most vulnerable groups that need immediate attention. When the factor of having more than two inpatient members combined with the characteristics related to low socioeconomic status, household size ≤ 2, the CHE rate of these families increased dramatically. Those household heads with characteristics of multiple vulnerabilities should be the priority intervention target, and related stakeholders should cooperate to establish a family information sharing platform and intervene in the issue based on cooperation.

## Data Availability Statement

The data analyzed in this study is subject to the following licenses/restrictions: Datasets used in this study are available from Centre of Health Statistics and Information, National Health Commission of the People's Republic of China. The data are not publicly available due to the confidential policy. Requests to access these datasets should be directed to the website of National Health Commission of the People's Republic of China: http://www.nhc.gov.cn/.

## Ethics Statement

The study involving human participants were reviewed and approved by ethics clearance was obtained from the Medical Ethics Committee at Harbin Medical University. The patients/participants provided their written informed consent to participate in this study.

## Author Contributions

JW, XT, and QW designed the study. LS contributed to data processing. JW, XQ, and HL contributed to result analysis. JW and XT drafted the manuscript. XZ, KW, SJ, QX, NM, and PC assisted with the collecting literature and providing suggestions for this manuscript. QW, YL, and ZK revised the paper. DA and BI provide great help with providing suggestions and collecting the literature during the revision. All authors contributed to the article and approved the submitted version.

## Conflict of Interest

The authors declare that the research was conducted in the absence of any commercial or financial relationships that could be construed as a potential conflict of interest.

## Publisher's Note

All claims expressed in this article are solely those of the authors and do not necessarily represent those of their affiliated organizations, or those of the publisher, the editors and the reviewers. Any product that may be evaluated in this article, or claim that may be made by its manufacturer, is not guaranteed or endorsed by the publisher.
