# Supplementary material for: Minimizing the Risk of Catastrophic Health Expenditure in China: A Multi-Dimensional Analysis of Vulnerable Groups
Source: Front Public Health. 2021 Aug 6;9:689809. doi: 10.3389/fpubh.2021.689809 (PMC8377675; doi:10.3389/fpubh.2021.689809)
Supplement: Supplementary file 1 [file Table_1.DOC]

**Table S1 CHE across consumption expenditure quintiles, areas, and** medical insurance types

|  |  | **Average monthly OOP（USD）** | **Average household’s capacity to pay（USD）** | **OOP to household’s capacity to pay（%）** |
| --- | --- | --- | --- | --- |
|  |
| **Areas** | **Eastern** | 52.2 | 350.5 | 17.12 |
|  | **central** | 46.2 | 262.0 | 18.49 |
|  | **Western** | 45.5 | 255.3 | 19.25 |
|  | **Urban** | 53.9 | 358.0 | 17.02 |
|  | **Rural** | 42.1 | 220.5 | 19.56 |
| **Expenditure quintile^c^** | 1 | 15.8 | 71.7 | 23.60 |
|  | 2 | 26.3 | 137.6 | 19.46 |
|  | 3 | 36.3 | 210.1 | 17.49 |
|  | 4 | 50.1 | 326.3 | 15.56 |
|  | 5 | 111.3 | 700.7 | 15.34 |
| **Medical insurance schemes** | **UEBMI** | 60.7 | 398.4 | 16.4 |
|  | **URBMI** | 48.6 | 270.7 | 18.7 |
|  | **NCMS** | 41.0 | 207.7 | 20.0 |
|  | **Integrated insurance^a^** | 47.7 | 307.6 | 18.1 |
|  | **Mixture^b^** | 51.5 | 460.7 | 13.4 |
|  | **Other types & none** | 42.7 | 311.7 | 15.1 |
|  | **National Average** | 41.3 | 249.1 | 18.3 |

**a: Integration insurance refers to other basic medical insurance types which integrate either URBMI and NCMS or UEBMI, URBMI and NCMS.**

**b: Mixture refers to participants covered by basic medical insurance and commercial insurance at the same time**

**c: Quintile 1 is the poorest 20%, and quintile 5 is the wealthiest 20%**

**USD: United States Dollar, according to the exchange rate of 6.1932 yuan to USD 1.00**
