# Supplementary figures and images for: Minimizing the Risk of Catastrophic Health Expenditure in China: A Multi-Dimensional Analysis of Vulnerable Groups
Source: Front Public Health. 2021 Aug 6;9:689809. doi: 10.3389/fpubh.2021.689809 (PMC8377675; doi:10.3389/fpubh.2021.689809)

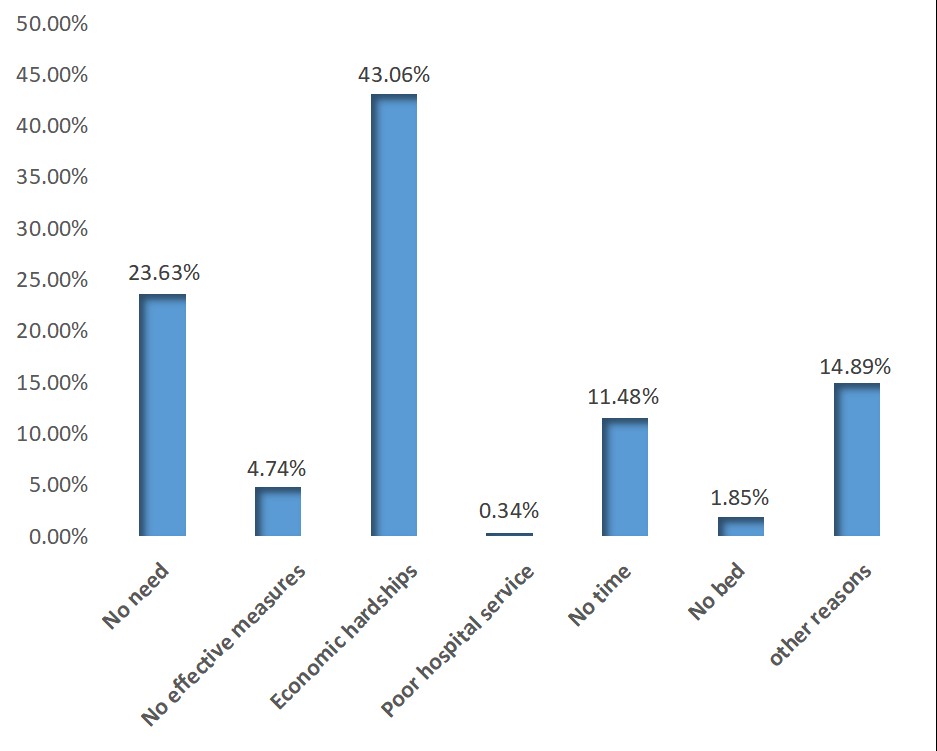


Figure S1 Reasons for not hospitalized despite the need for it

Supplement: Supplementary file 2 [file Table_2.DOC]
